# ABT‐199 (Venetoclax), a BH3‐mimetic Bcl‐2 inhibitor, does not cause Ca^2+^‐signalling dysregulation or toxicity in pancreatic acinar cells

**DOI:** 10.1111/bph.14505

**Published:** 2018-11-08

**Authors:** Monika A Jakubowska, Martijn Kerkhofs, Claudio Martines, Dimitar G Efremov, Julia V Gerasimenko, Oleg V Gerasimenko, Ole H Petersen, Geert Bultynck, Tim Vervliet, Pawel E Ferdek

**Affiliations:** ^1^ Medical Research Council Group, School of Biosciences Cardiff University Cardiff UK; ^2^ International Associated Laboratory (LIA), Malopolska Centre of Biotechnology Jagiellonian University Krakow Poland; ^3^ Laboratory of Molecular and Cellular Signaling, Department of Cellular and Molecular Medicine KU Leuven Leuven Belgium; ^4^ Molecular Hematology Unit International Centre for Genetic Engineering and Biotechnology Trieste Italy; ^5^ Department of Cell Biology, Faculty of Biochemistry, Biophysics and Biotechnology Jagiellonian University Krakow Poland

## Abstract

**Background and Purpose:**

Many cancer cells depend on anti‐apoptotic B‐cell lymphoma 2 (Bcl‐2) proteins for their survival. Bcl‐2 antagonism through Bcl‐2 homology 3 (BH3) mimetics has emerged as a novel anti‐cancer therapy. ABT‐199 (Venetoclax), a recently developed BH3 mimetic that selectively inhibits Bcl‐2, was introduced into the clinic for treatment of relapsed chronic lymphocytic leukaemia. Early generations of Bcl‐2 inhibitors evoked sustained Ca^2+^ responses in pancreatic acinar cells (PACs) inducing cell death. Therefore, BH3 mimetics could potentially be toxic for the pancreas when used to treat cancer. Although ABT‐199 was shown to kill Bcl‐2‐dependent cancer cells without affecting intracellular Ca^2+^ signalling, its effects on PACs have not yet been determined. Hence, it is essential and timely to assess whether this recently approved anti‐leukaemic drug might potentially have pancreatotoxic effects.

**Experimental Approach:**

Single‐cell Ca^2+^ measurements and cell death analysis were performed on isolated mouse PACs.

**Key Results:**

Inhibition of Bcl‐2 *via* ABT‐199 did not elicit intracellular Ca^2+^ signalling on its own or potentiate Ca^2+^ signalling induced by physiological/pathophysiological stimuli in PACs. Although ABT‐199 did not affect cell death in PACs, under conditions that killed ABT‐199‐sensitive cancer cells, cytosolic Ca^2+^ extrusion was slightly enhanced in the presence of ABT‐199. In contrast, inhibition of Bcl‐xL potentiated pathophysiological Ca^2+^ responses in PACs, without exacerbating cell death.

**Conclusion and Implications:**

Our results demonstrate that apart from having a modest effect on cytosolic Ca^2+^ extrusion, ABT‐199 does not substantially alter intracellular Ca^2+^ homeostasis in normal PACs and should be safe for the pancreas during cancer treatment.

**Linked Articles:**

This article is part of a themed section on Mitochondrial Pharmacology: Featured Mechanisms and Approaches for Therapy Translation. To view the other articles in this section visit http://onlinelibrary.wiley.com/doi/10.1111/bph.v176.22/issuetoc

**Abbreviations**
[Ca^2+^]_i_: intracellular cytosolic Ca^2+^ concentration
Bak: Bcl‐2 homologous antagonist killer
Bax: Bcl‐2‐associated X protein
Bcl‐2: B‐cell lymphoma 2
Bcl‐w: Bcl‐2‐like protein 2
Bcl‐xL: Bcl‐extra large
BH: Bcl‐2 homology
Bim: Bcl‐2‐like protein 11
CCK: cholecystokinin
CLL: chronic lymphocytic leukaemia
DLBCL: diffuse large B‐cell lymphoma
IP_3_R: inositol 1,4,5‐trisphosphate receptor
PAC: pancreatic acinar cell
PMCA: plasma membrane Ca^2+^ ATPase
RyR: ryanodine receptor
SERCA: sarco/endoplasmic reticulum Ca^2+^ ATPase
Tg: thapsigargin
TLC‐S: taurolithocholic acid 3‐sulfate

## Introduction

Impaired regulation of apoptosis is crucial to the process of carcinogenesis enabling cancer cells to evade cell death signals triggered by oncogenic stress and acquiring metastatic properties by accumulation of secondary genetic mutations (Adams and Cory, 2007; Hanahan and Weinberg, 2011). In cancer cells, this is achieved by altered expression levels of either the pro‐ or anti‐apoptotic B‐cell lymphoma 2 (**Bcl‐2**) family members, predominantly located at the mitochondrial membranes (Davids and Letai, 2012). Pro‐apoptotic Bcl‐2‐associated X protein (Bax) and Bcl‐2 homologous antagonist killer (Bak) are critical in the initiation of mitochondrial outer membrane permeabilization, the point of no return for apoptosis induction, whereas the anti‐apoptotic Bcl‐2 members [such as Bcl‐2, Bcl‐extra large (Bcl‐xL) or Bcl‐2‐like protein 2 (Bcl‐w)] counteract this process (Chipuk *et al.,* 2008). Bcl‐2‐dependent cancers are often ‘primed for death', a term used to describe the necessity of expressing high levels of the anti‐apoptotic Bcl‐2 proteins in order to actively sequester and inhibit the pro‐apoptotic family members, particularly Bax and the activator Bcl‐2 homology 3 (**BH3**)‐only protein Bcl‐2‐like protein 11 (Bim) (Akl *et al.,* 2014). Therefore, pharmacological disruption of the interaction between the anti‐ and pro‐apoptotic Bcl‐2 family members has the potential to activate Bax/Bak, and restore the programmed cell death (Wang *et al.,* 2000; Chipuk *et al.,* 2008). This spurred the development of Bcl‐2 antagonists that target the hydrophobic cleft of the anti‐apoptotic Bcl‐2 proteins displacing Bax/Bak and Bim from Bcl‐2. A very successful strategy has been the development of so‐called ‘BH3 mimetic' molecules that structurally resemble the BH3 domain of sensitizer BH3‐only proteins like Bad (Bcl‐2‐associated death promoter), thereby inhibiting Bcl‐2 without directly activating Bax/Bak (Oltersdorf *et al.,* 2005).

The early generation of BH3 mimetics, such as HA14‐1 and BH3I‐2′, despite being able to disrupt the interaction between the pro‐ and anti‐apoptotic proteins to initiate apoptosis (Wang *et al.,* 2000; Degterev *et al.,* 2001), had serious limitations that prevented their translation into the clinic. Some of these limitations have been linked to their adverse impact on the Ca^2+^ signalling machinery in non‐tumoural cells that is essential for intracellular Ca^2+^ homeostasis, cell function and survival (Vervloessem *et al.,* 2018). For instance, HA14‐1 also inhibits the Ca^2+^‐pump activity of the sarco/endoplasmic reticulum Ca^2+^ ATPase (SERCA), provoking cell death in part by depleting the ER Ca^2+^‐store (Hermanson *et al.,* 2009; Akl *et al.,* 2013). Moreover, both HA14‐1 and BH3I‐2′ were found to have pancreatotoxic effects (Gerasimenko *et al.,* 2010; Ferdek *et al.,* 2017). These effects were mediated directly by pathological Ca^2+^ responses in pancreatic acinar cells (PACs), the secretory epithelium that produces and releases digestive enzymes in the pancreas (Gerasimenko *et al.,* 2010). It is well established that excessive Ca^2+^ signals underlie the pathogenesis of acute pancreatitis, a severe human disease (Gerasimenko *et al.,* 2018). Moreover, dysregulation of Ca^2+^ homeostasis has also been implicated in the development of acute pancreatitis provoked by the anti‐leukaemic drug L‐asparaginase, which is used to treat childhood acute lymphoblastic leukaemia (Peng *et al.,* 2018; Vervliet *et al.,* 2018). In such cases, the anti‐cancer therapy must be ceased. At the mechanistic level, an excessive intracellular cytosolic Ca^2+^ concentration ([Ca^2+^]_i_) together with the depletion of intracellular Ca^2+^ stores in the ER and acidic pools, including zymogen granules, triggers premature protease activation *in situ* in PACs leading to autodigestion of the tissue (Petersen *et al.,* 2011).

Nevertheless, the development of BH3 mimetics continued, resulting in ABT‐737 and its orally available successor ABT‐263 (navitoclax), as selective on‐target inhibitors of Bcl‐2, Bcl‐xL and Bcl‐w that are able to induce cancer cell death (Oltersdorf *et al.,* 2005; Del Gaizo Moore *et al.,* 2008). Although these two pharmacological agents were shown to be effective at killing several Bcl‐2‐dependent cancer types (Oltersdorf *et al.,* 2005; Tse *et al.,* 2008), they were also found to induce thrombocytopenia and deregulate Ca^2+^ homeostasis in platelets, effects attributed to Bcl‐xL inhibition (Schoenwaelder *et al.,* 2011; Vogler *et al.,* 2011). Recently, ABT‐199 (venetoclax) has been developed (Souers *et al.,* 2013). This orally bioavailable selective inhibitor of Bcl‐2 was shown to cause cell death in chronic lymphocytic leukaemia (CLL) cells with an EC_50_ < 10 nM (Souers *et al.,* 2013). In 2016, ABT‐199 became the first ever clinically approved small molecule drug targeting a protein–protein interaction, and since then it has been used in the clinic as a therapy for relapsed CLL (Green, 2016). This drug is attracting a lot of interest and is currently undergoing further clinical trials, often in combination with other chemotherapeutic agents (Ferdek and Jakubowska, 2017).

Given that several of the early BH3 mimetics have been shown to alter intracellular Ca^2+^ signalling, particularly in the pancreas (Gerasimenko *et al.,* 2010; Ferdek *et al.,* 2017), it became essential and timely to determine whether or not the recently approved anti‐leukaemic drug ABT‐199 and other inhibitors such as selective Bcl‐xL antagonist A‐1155463 and Bcl‐2/Bcl‐xL inhibitor ABT‐737 show effects that might potentially be pancreatotoxic. Although ABT‐199 was shown not to dysregulate intracellular Ca^2+^ signalling upon acute application in the Bcl‐2‐dependent diffuse large Bcl (DLBCL) cell lines (Vervloessem *et al.,* 2017b, 2018), data on healthy primary cells are very limited. It is also unclear whether prolonged exposure to ABT‐199 could influence intracellular Ca^2+^ signalling in normal PACs. What is more, in PACs, physiological enzyme secretion is controlled by [Ca^2+^]_i_ oscillations triggered by ACh and cholecystokinin (CCK) (Petersen and Tepikin, 2008). Therefore, it is also important to assess whether these physiological Ca^2+^ signals are affected by ABT‐199. Furthermore, inducers of pancreatitis, such as certain bile acids, initiate pancreatic pathology *via* abnormal Ca^2+^ responses (Gerasimenko *et al.,* 2006; Ferdek *et al.,* 2016). However, potential synergistic effects between those inducers and ABT‐199, which may lead to aggravation of the disease, have not yet been addressed. ABT‐199 has already been introduced successfully into the clinic as an anti‐leukaemic agent, and its potential therapeutic applications are likely to increase in due course. Since acute pancreatitis is a side effect of some (5–10% of cases) existing therapies for acute lymphoblastic leukaemia in children (Kearney *et al.,* 2009; Raja *et al.,* 2014) and Ca^2+^ signals have been implicated in this process (Peng *et al.,* 2016), it has become particularly relevant to assess the impact of ABT‐199 (along with the selective Bcl‐xL inhibitor A‐1155463 and the Bcl‐2/Bcl‐xL inhibitor ABT‐737) on intracellular Ca^2+^ homeostasis and dynamics in healthy PACs.

## Methods

### Animals: Housing, husbandry and experimental procedures

Experimental animals (6‐week‐old male C57BL6/J mice, 23 ± 3 g) were purchased from Charles River UK and then housed in the Cardiff University or the Jagiellonian University institutional animal units (12 h light cycle) and maintained on a standard rodent chow diet with free access to water. Up to five mice were kept per cage with aspen wood bedding material and an enriched environment (cardboard tunnel, nesting material, wooden gnawing sticks, etc.). All procedures involving animals were performed in accordance with the UK Home Office or the Polish Ministry of Science and Higher Education regulations. The mice were humanely killed by cervical dislocation according to Schedule 1 of Animals (Scientific Procedures) Act 1986. The pancreatic tissue was removed for further experimental procedures. In order to reduce the number of animals needed for the experiments (in line with the 3Rs), cells isolated from one animal were used by two researchers simultaneously.

### Isolation of PACs

PAC isolation was performed as described previously (Ferdek *et al.,* 2017). The isolation and the experimental work was carried out in NaHEPES buffer. Unless otherwise stated, NaHEPES was supplemented with 1 mM Ca^2+^. Immediately after dissection, the pancreatic tissue was washed twice in NaHEPES, treated with collagenase (200 U·mL^−1^, in NaHEPES) and then enzymatically digested in the collagenase solution at 37°C for 15 min. After digestion, the pancreas was broken down by pipetting, suspended in NaHEPES, spun (1 min, 0.2 × *g*), resuspended in NaHEPES and spun again. Finally, isolated cells were suspended in NaHEPES and loaded with a Ca^2+^‐sensitive dye, Fluo‐4, as described below.

### Cytosolic Ca^2+^ measurements

Isolated PACs were loaded with 5 μM Fluo‐4 AM [30 min, at room temperature (RT)]. After the incubation, the cells were resuspended in fresh NaHEPES and used for experiments (RT) in a flow chamber perfused with NaHEPES‐based extracellular solution. Experiments were performed using the following equipment: (i) Zeiss LSM 880 confocal microscope (×63 oil objective) at the Jagiellonian University; and (ii) Leica TCS SPE confocal microscope (×63 oil objective) or Leica TCS SP5 II two‐photon confocal microscope (×63 water objective) at Cardiff University. Excitation was set to 488 nm and emission to 500–600 nm. Static images were taken at 512 × 512 pixel resolution, and a series of images were recorded at 256 × 256 pixel resolution; two consecutive frames were averaged, and time resolution was one image per 2 s. Fluorescence signals were plotted as F/F_0_, where F_0_ was an averaged signal from the first 10 baseline images.

### Cell death assay in PACs

Cell death assay was performed using an Annexin V‐FITC/PI apoptosis detection kit. PACs were isolated as described above, spun down and then suspended in 3 mL of fresh NaHEPES. The cells were divided equally into the experimental groups (final volume of NaHEPES: 2 mL) and kept until further treatment (4°C). Then 1 mL of the buffer was removed, and the cells were treated with 1 mL of 2× concentrated incubation buffer containing menadione or TLC‐S, and the vehicle (2 or 4 h, RT); the isolation quality control group was incubated with NaHEPES only. Also, in some treatment groups, the incubation buffer at this stage was supplemented with the BH3 mimetics: ABT‐199, A‐1155463, ABT‐199 together with A‐1155463, or ABT‐737. The final concentrations were as follows: menadione 60 μM and TLC‐S 200 μM; and the BH3 mimetics: 1 μM ABT‐199, 1 μM A‐1155463, 1 μM ABT‐199 together with 1 μM A‐1155463, or 10 μM ABT‐737. Owing to the limited amount of mouse PACs obtained in a single isolation procedure, all experimental groups but one were equal by design. For the cell death assay, the cells isolated from 16 pancreata were either treated with NaHEPES (the isolation quality control, *N* = 16) or randomized to the experimental groups with five independent pancreata per group (*N* = 5). Since the NaHEPES‐treated sample served as the control for each individual pancreas isolation (16 in total) subsequently used in the cell death experiments, *N* was 16 for this condition. Fifteen minutes before the end of the incubation, Annexin V‐FITC and PI were added to the samples. The cells were visualized with a TCS SP5 II two‐photon confocal microscope (Leica) with a 63× 1.2 NA water objective, and fluorescence/transmitted light images were taken. Annexin‐V‐FITC (excitation: 488 nm, emission: 510–555 nm) specifically stains apoptotic cells, whereas PI (excitation: 535 nm, emission: 585–650 nm) was used for detection of necrotic cells; the cells stained with both fluorescent dyes were classified as secondary necrosis. Fifteen pictures of independent cell clusters were taken at 512 × 512 pixel resolution. The percentage of live, apoptotic, secondary necrotic and necrotic cells were counted in each treatment group by one researcher in a blinded fashion (encoding the group labels).

### Cell death assay in B‐cell lymphoma lines and CLL patient samples

DLBCL cell lines were seeded at 250 000 cells·mL^−1^ 24 h before treatment. Cells were harvested at 2, 4 and 6 h after 1 μM ABT‐199 or vehicle treatment and stained with Alexa Fluor™ 488 Annexin V/7‐AAD. Flow cytometry was used for data acquisition (Attune; Thermo Fisher Scientific) whereby viable cells were identified as being Annexin V/7‐AAD negative. The analysis was performed using the FlowJo software.

Blood samples were collected from patients with CLL according to the principles established by the International Conference on Harmonization Guidelines on Good Clinical Practice. An informed consent was obtained from all patients and approval for the study was obtained from the ethical committee of the Università Cattolica del Sacro Cuore, Fondazione Policlinico A. Gemelli, Rome, Italy (protocol number 14563/15). The collection and analysis of CLL patient samples were performed as reported in Bojarczuk *et al*. (2016). Briefly, mononuclear cells were isolated from peripheral blood samples by Ficoll gradient centrifugation. The proportion of CD5^+^CD19^+^ CLL cells was determined by flow cytometry; samples containing >85% CLL cells were used for the subsequent experiments. CLL cells were cultured at a cell density of 1 × 10^7^ mL^−1^ in RPMI 1640 supplemented with 10% heat‐inactivated FBS, 100 U·mL^−1^ penicillin, 0.1 mg·mL^−1^ streptomycin, 2 mM L‐glutamine and 1 mM sodium pyruvate (Invitrogen) and treated for 2, 4 or 6 h with ABT‐199 (1 μM) or vehicle. The percentage of apoptotic cells was determined by staining with PI and annexin‐A5‐FITC conjugate (Nexins Research) and analysis on an FACSCalibur flow cytometer with CellQuest Pro version 5.2.1 software (BD Biosciences).

### Comparison of Ca^2+^ extrusion

In order to empty the ER Ca^2+^ stores, PACs were treated with 2 μM **thapsigargin** (Tg) for 10 min in the absence of extracellular Ca^2+^. Then the extracellular Ca^2+^ concentration was increased to 10 mM, which induced Ca^2+^ influx to the cytosol. Once a [Ca^2+^]_i_ plateau was achieved, removal of extracellular Ca^2+^ unmasked the process of Ca^2+^ extrusion across the plasma membrane. This phase of the response was further analysed and compared between control and ABT‐199 pretreated PACs. First, for every recorded [Ca^2+^]_i_ trace, the maximum and minimum F/F_0_ values in the rage of 1000–1400 s of the response were determined (F_max_ and F_min_). The normalized fluorescence that corresponds to half the decrease between these two values was calculated as follows: F_1/2_ = F_min_ + (F_max_−F_min_)/2. Next, a linear fit to the extrusion phase was determined. Time values corresponding to F_max_ (t_max_) and F_1/2_ (t_1/2_) were calculated from the linear fit. Finally, t_1/2_ was calculated as the difference between t(F_1/2_) and t_max_. The t_1/2_ values obtained for control and ABT‐199‐treated cells were then averaged and presented as dot charts ±SEM. Student's *t*‐test was applied for statistical comparison, and the significance threshold was set at 0.05.

### Statistical analysis

The data and statistical analysis comply with the recommendations on experimental design and analysis in pharmacology (Curtis *et al.,* 2015). At least five independent repeats (*N* = 5) were performed in each experimental setting (see the text for details). Additionally, in Ca^2+^ signalling experiments, *n* values representing the recorded fluorescence of the specific regions of interest (ROI), corresponding to single cells, were provided. Those were not the technical replicates but the independent measurements of the entire cell population in the experiment. Because of the non‐equal numbers cells recorded in the viewing fields, *n* may vary between treatment groups in the given experimental setting. Quantitative analysis of Ca^2+^ responses was performed as described previously (Ferdek *et al.,* 2017). Briefly, areas under the individual traces were calculated according to the formula: Σ(F/F_0_−F_0_) × Δt, where F is the recorded fluorescence, F_0_ is the baseline fluorescence and Δt is the time interval. The values obtained were then averaged and presented as dot charts ±SEM. Student's *t*‐test was applied for statistical comparison, and the significance threshold was set at 0.05. Quantitative analysis of cell death was performed using SPSS Statistics 24 software (IBM): first, Levene's test was used to assess the equalities of the variances for a variable calculated for the groups, and then ANOVA or a non‐parametric Kruskal–Wallis one‐way ANOVA were applied to compare the differences among group means; for both tests, the significance thresholds were set at 0.05. Finally, Bonferroni's *post hoc* test (whenever relevant) was performed only if values of the *F*‐test (used to assess the equalities of the means of a given set of normally distributed populations, all having the same SD) achieved the necessary level of statistical significance (*P* < 0.05).

### Materials

The main reagents for cell isolation and imaging included: Annexin V‐FITC/propidium iodide (PI) apoptosis detection kit, Fluo‐4 AM (Thermo Fisher Scientific, Loughborough, UK); collagenase (Worthington, Lakewood, USA); ACh, CCK, inorganic salts, menadione, **taurolithocholic acid 3‐sulfate (TLC‐S)** (Sigma‐Aldrich, Dorset, UK); and the BH3 mimetics: the Bcl‐2 inhibitor ABT‐199 (Active Biochem, Bonn, Germany), the Bcl‐xL inhibitor A‐1155463 (Selleckchem, Cambridgeshire, UK) and the non‐selective Bcl‐2, Bcl‐xL and Bcl‐w inhibitor ABT‐737 (Santa Cruz Biotechnology, Heidelberg, Germany). NaHEPES buffer was prepared as follows (mM): NaCl 140, KCl 4.7, HEPES 10, MgCl_2_ 1, glucose 10; pH 7.3. The BH3 mimetics were dissolved in DMSO, TLC‐S in NaHEPES and menadione in ethanol.

### Nomenclature of targets and ligands

Key protein targets and ligands in this article are hyperlinked to corresponding entries in http://www.guidetopharmacology.org, the common portal for data from the IUPHAR/BPS Guide to PHARMACOLOGY (Harding *et al.,* 2018), and are permanently archived in the Concise Guide to PHARMACOLOGY 2017/18 (Alexander *et al.,* 2017a, 2017b, 2017c, 2017d).

## Results

### Selective inhibition of only one Bcl‐2 family member by BH3 mimetics does not induce noxious Ca^2+^ responses

**Figure 1:**
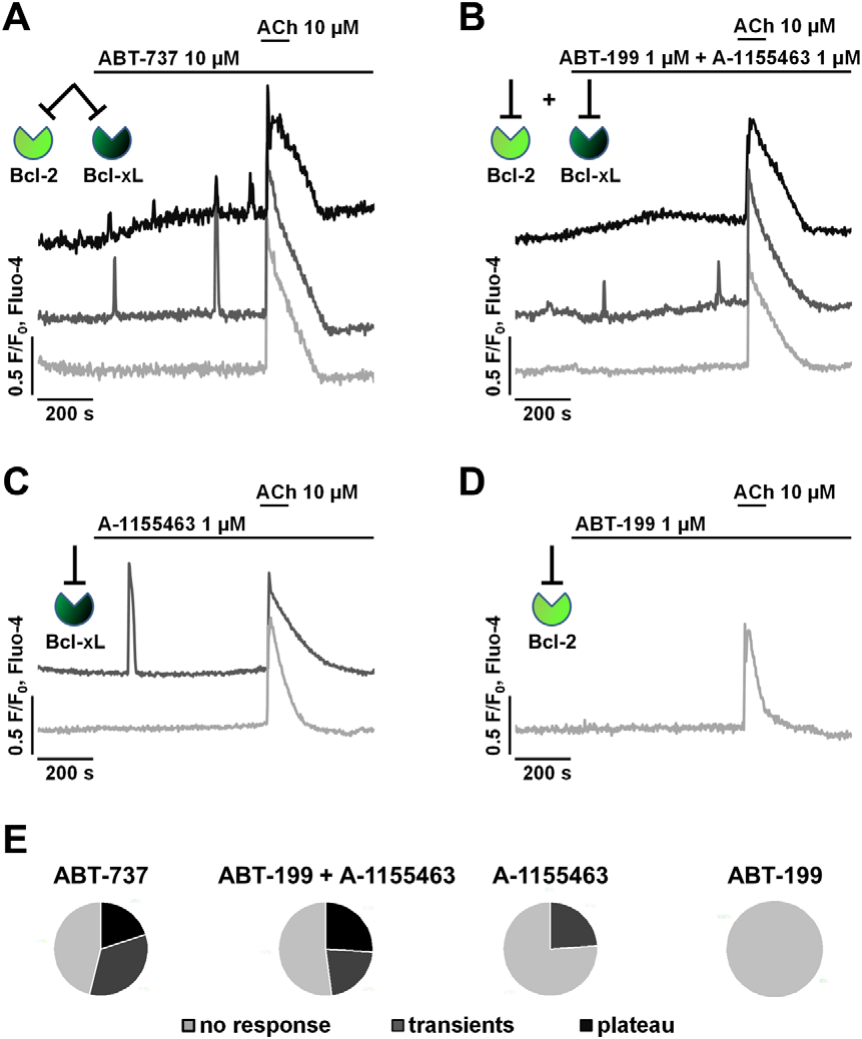
Selective pharmacological inhibition of single anti‐apoptotic Bcl‐2 family members does not induce substantial Ca^2+^ responses in mouse PACs. ACh at supramaximal concentration (10 μM) triggers Ca^2+^ release from the ER and thus can be used to identify responding/live cells. Only PACs that responded to ACh were included in the analysis of these experiments. Green insets illustrate which Bcl‐2 family proteins are targeted by the used inhibitors in each figure panel. *N*, number of independent repeats in the experiments; *n*, number of independent regions of interest in the experiment (see the text for details). (A) Sample traces (*N* = 7, *n* = 80) show three patterns of [Ca^2+^]_i_ responses recorded in PACs upon acute application of 10 μM ABT‐737, an inhibitor of Bcl‐2 and Bcl‐xL (as indicated in the figure); 46 % of cells did not show any Ca^2+^ response (light grey), 34% of cells responded with one or more Ca^2+^ transients (dark grey), and 20% of cells developed an increased cytosolic Ca^2+^ plateau (black). (B) Sample traces (*N* = 6, *n* = 46) show three patterns of [Ca^2+^]_i_ responses recorded in PACs upon acute application of 1 μM ABT‐199 (selective Bcl‐2 inhibitor) together with 1 μM A‐1155463 (selective Bcl‐xL inhibitor); 52 % of cells did not show any Ca^2+^ response (light grey), 22% of cells responded with one or more Ca^2+^ transients (dark grey), and 26% of cells developed an increased cytosolic Ca^2+^ plateau (black). (C) Sample traces (*N* = 6, *n* = 41) show two patterns of [Ca^2+^]_i_ responses recorded in PACs upon acute treatment with 1 μM A‐1155463. In the majority of cells (76%), A‐115546 did not cause any Ca^2+^ response (light grey), whereas in approximately 24% of cells, single Ca^2+^ transients were recorded (dark grey). (D) Sample trace (*N* = 5, *n* = 52) shows lack of Ca^2+^ response to 1 μM ABT‐199 in PACs (light grey). (E) Summary of [Ca^2+^]_i_ response patterns in PACs to different inhibitors of Bcl‐2 family members, colour coding as above. The selective pharmacological Bcl‐2 inhibitor ABT‐199 does not have a major effect on intracellular Ca^2+^ in acinar cells.

In order to compare the effects of acute inhibition of different Bcl‐2‐protein family members on Ca^2+^ homeostasis in PACs, several BH3 mimetics were tested (Figure 1). First, 10 μM ABT‐737 was used to target the hydrophobic clefts of a wide range of Bcl‐2 proteins: Bcl‐2, Bcl‐xL and Bcl‐w (Figure 1A). Further pharmacological inhibition of two anti‐apoptotic Bcl‐2 family members, Bcl‐2 and Bcl‐xL, was achieved by the combination of two selective inhibitors at 1:1 ratio, 1 μM ABT‐199 and 1 μM A‐1155463 (Figure 1B). These selective inhibitors were then tested individually (Figure 1C, D). Given the higher affinity of ABT‐199 and A‐1155463 for the respective hydrophobic clefts of Bcl‐2 and Bcl‐xL, compared to ABT‐737 (Konopleva *et al.,* 2006; Souers *et al.,* 2013; Tao *et al.,* 2014), these two inhibitors were used at lower concentrations (1 μM vs. 10 μM). Intracellular Ca^2+^ signals were recorded in PACs loaded with Ca^2+^‐sensitive fluorescent probe Fluo‐4. After the baseline signal had been recorded for 200 s, the cells were treated with the indicated BH3 mimetics for 600 s. Finally, ACh at supramaximal concentration (10 μM) was used as a positive control for ER Ca^2+^‐store loading.

Three patterns of Ca^2+^ responses occurred in PACs treated with ABT‐737 (Figure 1A). While 46% of cells did not show any Ca^2+^ response to the mimetic (but responded to ACh; light grey), 34% produced one or more Ca^2+^ transients (dark grey) and 20% of cells developed a prolonged cytosolic Ca^2+^ plateau (black). Interestingly, a similar response pattern was obtained when a combination of two selective inhibitors, ABT‐199 together with A‐1155463, was used (Figure 1B). In this case, while 52% of cells did not develop any Ca^2+^ responses to the inhibitors (but responded to ACh; light grey), 22% produced one or more Ca^2+^ transients (dark grey) and 26% developed an increased cytosolic Ca^2+^ plateau (black). Finally, selective inhibition of a single Bcl‐2‐family member was tested in PACs. Blocking Bcl‐xL with A‐1155463 did not trigger any Ca^2+^ responses in the majority of cells tested (76%, light grey), whereas 24% responded with one or more Ca^2+^ transients (light grey; Figure 1C). Importantly, ABT‐199, designed to selectively target Bcl‐2, did not elicit any Ca^2+^ signals in PACs (light grey; Figure 1D). Even when ABT‐199 was applied at a high dose (10 μM), the vast majority of cells (89%) did not develop Ca^2+^ responses and the remaining 11% of cells responded only with minor oscillations (not shown). Circular diagrams summarizing Ca^2+^ response patterns in PACs to different inhibitors of Bcl‐2 family members are shown in Figure 1E (colour coding corresponds with the traces). Taken together, these data suggest that selective inhibition of one Bcl‐2 family member does not induce substantial increases in intracellular Ca^2+^ (Figure 1C–E). In contrast, when more than one Bcl‐2 family protein is targeted by a BH3 mimetic, the intracellular Ca^2+^ homeostasis is influenced to a much greater extent (Figure 1A, B, E).

### ABT‐199, a selective inhibitor of Bcl‐2, does not alter physiological Ca^2+^ responses in PACs

**Figure 2:**
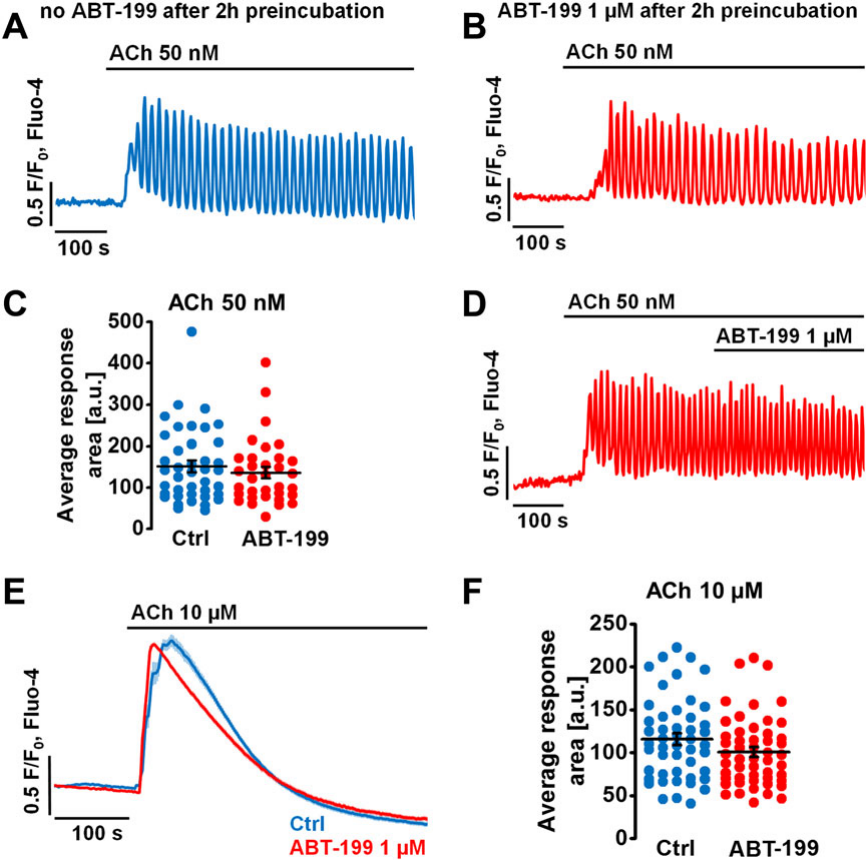
ABT‐199 does not affect physiological Ca^2+^ responses to ACh in mouse PACs. *N*, number of independent repeats in the experiments; *n*, number of independent regions of interest in the experiment (see the text for details). (A) Sample trace (*N* = 5, *n* = 40) shows physiological [Ca^2+^]_i_ oscillations in response to 50 nM ACh recorded after 2 h incubation in the extracellular buffer without ABT‐199. (B) Sample trace (*N* = 5, *n* = 33) shows physiological [Ca^2+^]_i_ oscillations in response to 50 nM ACh recorded after 2 h incubation with 1 μM ABT‐199. (C) Responses to ACh are quantitatively analysed by comparing the average [Ca^2+^]_i_ increase above the baseline levels recorded for 600 s post‐treatment: control (blue, *N* = 5, *n* = 40, 151.8 ± 14.1 a.u.) or in the presence of 1 μM ABT‐199 (red, *N* = 5, *n* = 33, 136.2 ± 13.9 a.u.). The responses are unaffected by ABT‐199. (D) Sample trace (*N* = 5, *n* = 33) shows that physiological [Ca^2+^]_i_ oscillations evoked by 50 nM ACh are unaffected by acute application of 1 μM ABT‐199. (E) Average traces show [Ca^2+^]_i_ responses in PACs to a supramaximal dose of ACh (10 μM) in the absence of extracellular Ca^2+^. Cells were incubated for 2 h in the absence (blue trace, *N* = 6, *n* = 48) or presence (red trace, *N* = 7, *n* = 51) of 1 μM ABT‐199. (F) Average traces shown in (E) are quantitatively analysed by comparing the areas under the traces recorded for 200 s post‐treatment: 10 μM ACh alone (blue, *n* = 48, 115.8 ± 6.8 a.u.) and ACh with ABT‐199 (red, *n* = 51, 100.8 ± 5.7 a.u.). Student's *t*‐test was applied for statistical analysis.

**Figure 3:**
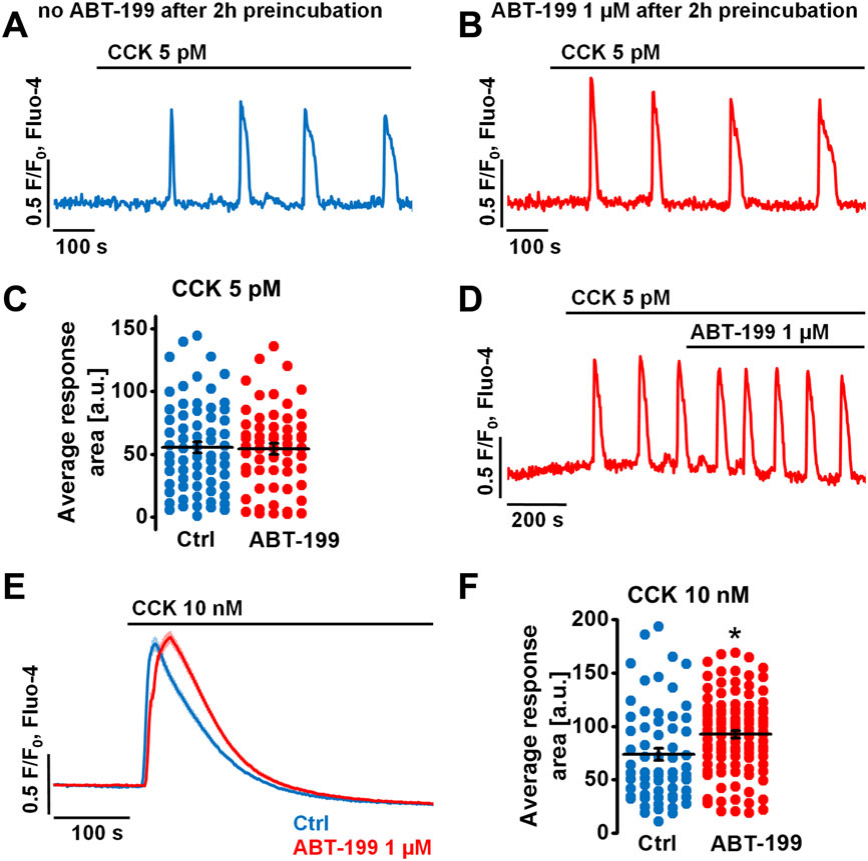
ABT‐199 does not affect physiological Ca^2+^ responses to CCK in mouse PACs. *N*, number of independent repeats in the experiments; *n*, number of independent regions of interest in the experiment (see the text for details). (A) Sample trace (*N* = 7, *n* = 66) shows [Ca^2+^]_i_ responses to 5 pM CCK recorded after 2 h incubation in the extracellular buffer without ABT‐199. (B) Sample trace (*N* = 8, *n* = 58) shows physiological [Ca^2+^]_i_ oscillations in response to 5 pM CCK recorded after 2 h incubation with 1 μM ABT‐199. (C) Responses to 5 pM CCK are quantitatively analysed by comparing the average [Ca^2+^]_i_ increase above the baseline levels recorded for 800 s post‐treatment: control (blue, *N* = 7, *n* = 66, 55.4 ± 4.5 a.u.) or in the presence of 1 μM ABT‐199 (red, *N* = 8, *n* = 58, 54.3 ± 4.3 a.u.). The responses are unaffected by ABT‐199. (D) Sample trace (*N* = 5, *n* = 100) shows that physiological [Ca^2+^]_i_ oscillations evoked by 5 pM CCK are unaffected by acute application of ABT‐199. (E) Average traces show [Ca^2+^]_i_ responses in PACs to a supramaximal dose of CCK (10 nM) in the absence of extracellular Ca^2+^. Cells were incubated for 2 h in the absence (blue trace, *N* = 5, *n* = 61) or presence (red trace, *N* = 8, *n* = 102) of 1 μM ABT‐199. (F) Average traces shown in (E) are quantitatively analysed by comparing the areas under the traces recorded for 200 s post‐treatment: 10 nM CCK alone (blue, *N* = 5, *n* = 61, 73.8 ± 5.7 a.u.) and CCK with ABT‐199 (red, *N* = 8, *n* = 102, 92.5 ± 3.6 a.u.). Student's *t*‐test was applied for statistical analysis.

ABT‐199 has recently been approved by the US Food and Drug Administration as an anti‐leukaemic agent. Therefore, identification of its potential side effects prior to more general clinical use is of particular importance. ABT‐199 at a low nM concentration (<10 nM) was shown to induce apoptosis in CLL cells (Souers *et al.,* 2013). Here, we used ABT‐199 at a much higher concentration (1 μM) to test whether it would affect the oscillatory Ca^2+^ signals that normally control physiological enzyme secretion in PACs (Figures 2 and 3; see also Figure 1D, E). Alternations in Ca^2+^ signals towards more global and sustained Ca^2+^ responses could indicate a serious risk of premature enzyme activation *in situ* in PACs and thus a considerable threat of autodigestion and necrosis of the pancreas, which may develop into acute pancreatitis (Petersen *et al.,* 2011; Gerasimenko *et al.,* 2014).

Single cell Ca^2+^ measurements were performed as described above. In PACs, ACh at nM concentrations triggers Ca^2+^ oscillations, primarily initiated by the IP_3_Rs and further amplified by the ryanodine receptors (RyRs) (Wakui *et al.,* 1990; Cancela, 2001). Potential effects of ABT‐199 on ACh‐elicited Ca^2+^ release in PACs were assessed in different experimental settings (Figure 2). First, the influence of prolonged incubation (2 h) with ABT‐199 on 50 nM ACh‐evoked oscillatory Ca^2+^ responses was tested and compared to untreated control. No differences were revealed between the control (Figure 2A, blue) and the ABT‐199‐treated PACs (Figure 2B, red); see also data representing individual areas under the traces (Figure 2C). In addition, acute application of ABT‐199 on top of ACh‐elicited Ca^2+^ oscillations neither affected the frequencies of the responses nor changed their amplitudes (Figure 2D). Finally, prolonged incubation (2 h) with ABT‐199 did not alter the global Ca^2+^ responses to ACh at supramaximal concentration (10 μM) in PACs (Figure 2E, F).

CCK, at physiologically relevant concentrations (low pM), induces Ca^2+^ transients in PACs, which are mediated predominantly by the RyRs (Thorn *et al.,* 1994; Cancela, 2001). To test whether ABT‐199 alters CCK‐elicited responses in PACs (Figure 3), a similar experimental approach as above was adopted. First, the influence of a prolonged incubation (2 h) with ABT‐199 on 5 pM CCK‐elicited oscillatory Ca^2+^ responses was tested and compared to an untreated control. Importantly, no differences were revealed between the control (Figure 3A, blue) and the ABT‐199‐treated PACs (Figure 3B, red); see also data representing individual areas under the traces (Figure 3C). Next, when ABT‐199 was applied acutely on top of CCK‐elicited Ca^2+^ oscillations, neither the frequencies nor the amplitudes of the responses were affected (Figure 3D). Prolonged incubation (2 h) with ABT‐199 (Figure 3E) slightly increased the global Ca^2+^ releases elicited by CCK at a supramaximal concentration (10 nM) in PACs (Figure 3E, F). Taken together, these results indicate that ABT‐199 does not substantially affect physiological Ca^2+^ responses in PACs.

### Inhibitors of Bcl‐2 family members affect pathophysiological Ca^2+^ responses in PACs

BH3 mimetics have been developed in order to counteract the Bcl‐2‐dependent evasion of apoptosis, common in cancers. Normal cells, such as PACs, are much less dependent on anti‐apoptotic Bcl‐2 family members than cancer cells. However, even in non‐transformed cells, pathophysiological stimulants that trigger cell death could influence the balance between the pro‐ and anti‐apoptotic Bcl‐2 proteins and thus may ‘prime these cells for death'. Therefore, pathophysiological stress may reveal additional effects of a BH3 mimetic that do not surface under physiological conditions.

**Figure 4:**
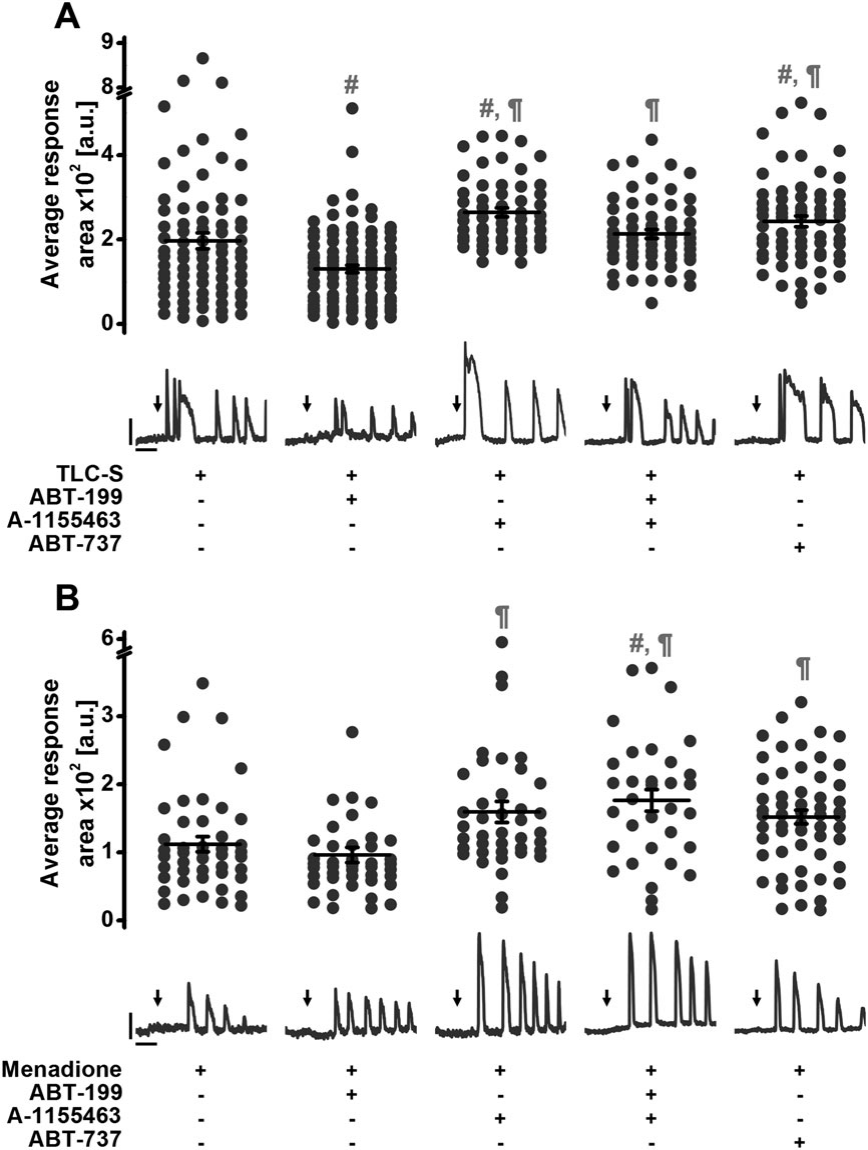
ABT‐199 does not potentiate pathophysiological Ca^2+^ responses evoked by TLC‐S or menadione. *N*, number of independent repeats in the experiments; *n*, number of independent regions of interest in the experiment (see the text for details). (A) Dot chart shows response areas calculated for the first 1000 s of [Ca^2+^]_i_ responses (and corresponding traces below show typical [Ca^2+^]_i_ responses) recorded in mouse PACs to 200 μM TLC‐S alone (Ctrl *N* = 6, *n* = 78, 196.8 ± 18.8 a.u.) or in the presence of 1 μM ABT‐199 (*N* = 6, *n* = 93, 130.3 ± 10.0 a.u.), 1 μM A‐1155463 (*N* = 5, *n* = 55, 264.2 ± 10.4 a.u.), 1 μM ABT‐199 together with 1 μM A‐1155463 (*N* = 5, *n* = 61, 213.1 ± 10.4 a.u.) or 10 μM ABT‐737 (*N* = 5, *n* = 65, 243.0 ± 12.9 a.u.). Cells were incubated for 2 h in the buffer with or without the appropriate inhibitor/s. One‐way ANOVA together with the Bonferroni's *post hoc* test were used for the statistical analysis; #, significant versus treatment with TLC‐S; ¶, significant versus treatment with TLC‐S + ABT‐199. (B) Dot chart shows response areas calculated for the first 1000 s of [Ca^2+^]_i_ responses (and corresponding traces below show typical [Ca^2+^]_i_ responses) recorded in mouse PACs to 60 μM menadione alone (Ctrl *N* = 6, *n* = 45, 112.0 ± 11.2 a.u.) or in the presence of 1 μM ABT‐199 (*N* = 5, *n* = 42, 96.2 ± 11.1 a.u.), 1 μM A‐1155463 (*N* = 5, *n* = 40, 159.5 ± 15.7 a.u.), 1 μM ABT‐199 together with 1 μM A‐1155463 (*N* = 5, *n* = 33, 176.4 ± 16.0 a.u.) or 10 μM ABT‐737 (*N* = 6, *n* = 56, 151.9 ± 10.1 a.u.). Cells were incubated for 2 h in the buffer with or without the appropriate inhibitor/s. Scale bars for both: vertical 0.5 F/F_0_ and horizontal 200 s. Kruskal–Wallis one‐way ANOVA (a nonparametric test) was used here for statistical analysis; #, significant versus treatment with menadione; ¶, significant versus treatment with menadione + ABT‐199.

To test whether inhibitors of Bcl‐2 proteins would modulate Ca^2+^ signals in PACs under a pathophysiological stimulus, the cells were incubated (2 h) with different BH3 mimetics (concentrations as in Figure 1) and then treated acutely with TLC‐S or menadione. Single‐cell Ca^2+^ measurements in PACs were performed as described above. The bile acid TLC‐S, known to induce pathophysiological Ca^2+^ signals and necrosis in PACs (Gerasimenko *et al.,* 2006), was used here as an initiator of necrotic stress (Figure 4A). An apoptotic stimulus was supplied by menadione, an agent with vitamin K activity, which triggers mitochondrial stress *via* excessive production of ROS (Figure 4B) (Monks *et al.,* 1992; Gerasimenko *et al.,* 2002). Menadione was also shown to elicit pathophysiological Ca^2+^ signals in PACs, associated with the induction of apoptosis (Gerasimenko *et al.,* 2002).

Substantial elevations in cytosolic Ca^2+^ in PACs were induced by 200 μM TLC‐S (Figure 4A). These elevations were reduced by the Bcl‐2 inhibitor ABT‐199 (*P* < 0.05), whereas A‐1155463 (blocker of Bcl‐xL) potentiated TLC‐S‐elicited Ca^2+^ release (*P* < 0.05). Interestingly, these two opposite effects cancelled out when ABT‐199 and A‐1155463 were applied simultaneously. Under the inhibition of a wide range of Bcl‐2 family proteins by ABT‐737, TLC‐S‐induced Ca^2+^ responses were potentiated (*P* < 0.05).

A similar experimental approach was used to test the effects of BH3 mimetics on 60 μM menadione‐elicited Ca^2+^ elevations in PACs (Figure 4B). ABT‐199 alone, despite failing to cause a clear reduction in menadione‐driven Ca^2+^ responses, definitely did not potentiate them. In contrast, A‐1155463 (when applied on its own or simultaneously with ABT‐199), as well as ABT‐737, both increased the pathophysiological Ca^2+^ elevations induced by menadione (*P* < 0.05). Taken together, pharmacological inhibition of Bcl‐xL *via* its hydrophobic cleft appears to increase pathophysiological Ca^2+^ responses, whereas inhibition of Bcl‐2 does not have a major impact on Ca^2+^ homeostasis both in physiology and pathology. Since ABT‐199 does not exacerbate pathophysiological Ca^2+^ signals, it may have a more advantageous safety profile compared to inhibitors that target Bcl‐xL.

### BH3 mimetics do not aggravate apoptotic or necrotic cell death in PACs

**Figure 5:**
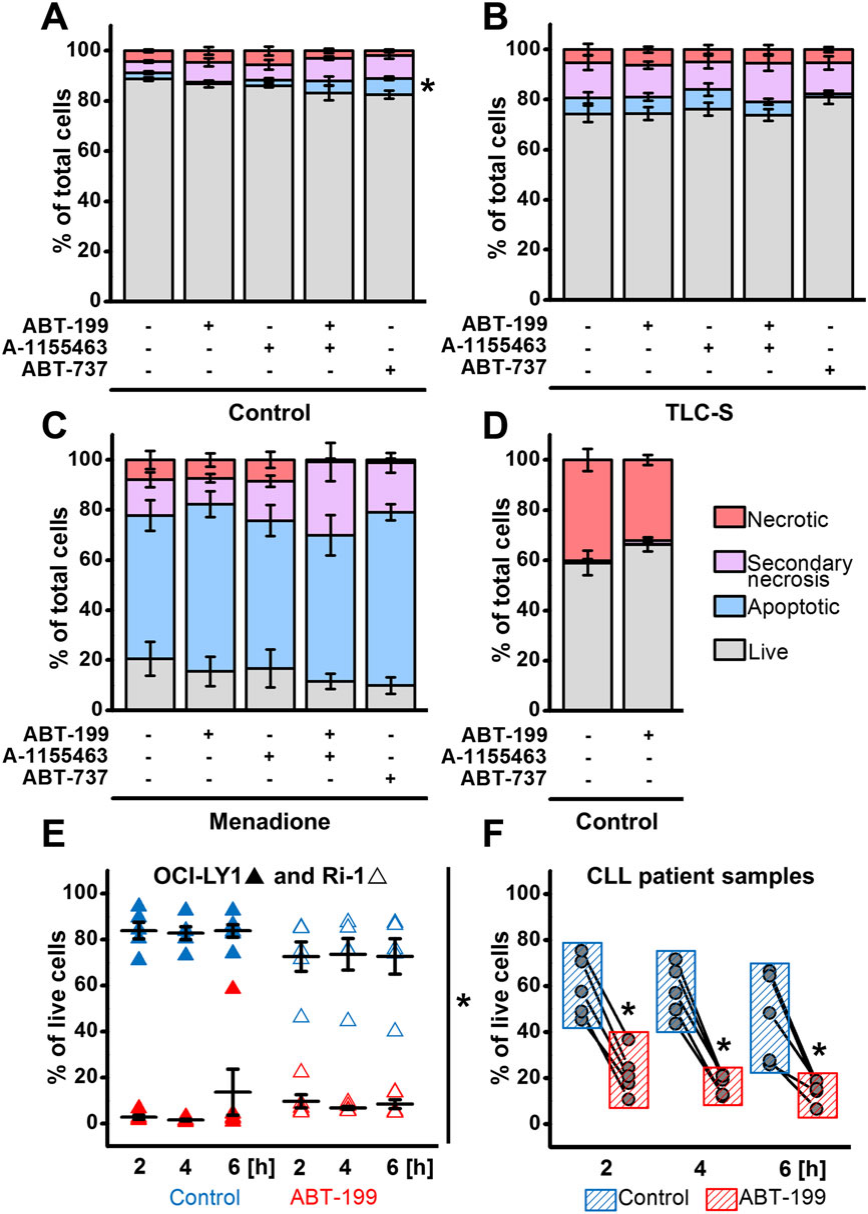
ABT‐199 applied under conditions capable of inducing cell death in Bcl‐2‐dependent cancer cells neither triggers nor potentiates cell death in PACs. (A–C) Bar charts (mean ± SEM) showing cell death in the absence of an additional trigger (A), induced by 2 h treatment with 200 μM TLC‐S (B) or induced by 2 h treatment with 60 μM menadione (C) in mouse PACs in the presence or absence of 1 μM ABT‐199, 1 μM A‐1155463, 1 μM ABT‐199 together with 1 μM A‐1155463 or by 10 μM ABT‐737 (*N* = 5, *n* = 75). Grey bars represent live cells; blue, apoptotic cells; pink, secondary necrosis; and red, necrosis. Kruskal–Wallis one‐way ANOVA (a nonparametric test) was used here for statistical analysis; *P < 0.05, significant versus treatment with ABT‐199 (apoptosis only). (D) Bar chart (mean ± SEM) showing cell death assessed after 4 h incubation with or without 1 μM ABT‐199 (*N* = 5); colour coding and statistical analysis as above. (E) Dot chart showing the percentage of live human DLBCL cell lines (OCI‐LY1 and Ri‐1) in the presence or absence of 1 μM ABT‐199 (*N* = 5) after 2, 4 or 6 h. Student's *t*‐test was applied for statistical analysis; *P < 0.05. (F) Before–after scatter plots showing the percentage of live CD5^+^CD19^+^ cells obtained from CLL patients, in the presence or absence of 1 μM ABT‐199 (*N* = 5) following a 2, 4 or 6 h incubation. Student's *t*‐test was applied for statistical analysis; *P < 0.05.

As shown above, the new generation of BH3 mimetics may alter TLC‐S‐ or menadione‐elicited Ca^2+^ responses in PACs. Since Ca^2+^ release is associated with the induction of necrosis and apoptosis, application of Bcl‐2 protein inhibitors may modulate PAC death. To determine this, PACs were incubated for 2 h with either TLC‐S (200 μM) or menadione (60 μM) in the presence of BH3 mimetics or appropriate vehicles (Figure 5A–C). Annexin V‐FITC/PI staining was used to assess the extent of cell death. The number of live (grey), apoptotic (blue), necrotic (red) cells as well as secondary necrosis (pink) was determined and is presented in Figure 5. Importantly, treatment with the selective BH3 mimetics did not induce cell death in PACs, indicating that dependence on Bcl‐2/Bcl‐xL for survival is rather limited in these cells (Figure 5A). Also, the presence of BH3 mimetics did not significantly alter TLC‐S‐ (Figure 5B) or menadione‐induced (Figure 5C) cell death in PACs.

We further scrutinized the effects of ABT‐199, given its use in clinical settings. First, a longer (4 h) incubation with ABT‐199 (1 μM) did not potentiate the increased cell death that spontaneously occurred in PACs maintained *in vitro* for longer periods (Figure 5D). Second, we also determined whether the ABT‐199 concentration (1 μM) and duration of the treatment (2 h) used in the PAC experiments could kill effectively Bcl‐2‐dependent cancer cells known to be sensitive to ABT‐199 (Anderson *et al.,* 2016; Vervloessem *et al.,* 2017a). For this, two DLBCL cell lines, OCI‐LY‐1 and Ri‐1, and primary CLL lymphocytes obtained from five different patients, were treated with 1 μM of ABT‐199 for 2, 4 or 6 h (Figure 5E, F). The number of living cells was determined using Alexa Fluor™ 488 Annexin V/7‐AAD or FITC Annexin V/PI staining. In all cell lines and patient samples tested, a 2 h treatment with 1 μM ABT‐199 showed extensive cell death that was not dramatically increased by extending the duration of the treatment. This suggests a near maximal effect of ABT‐199 at 1 μM in these cancer cells already after 2 h. Taken together, these results indicate that selective Bcl‐2 protein family inhibitors neither provoke cell death by themselves nor aggravate cell death induced by pathophysiological stimuli in PACs. For ABT‐199, we validated that the concentrations/time periods used were capable of inducing cell death in Bcl‐2‐dependent cancers.

### Selective inhibition of Bcl‐2 affects cytosolic Ca^2+^ extrusion

**Figure 6:**
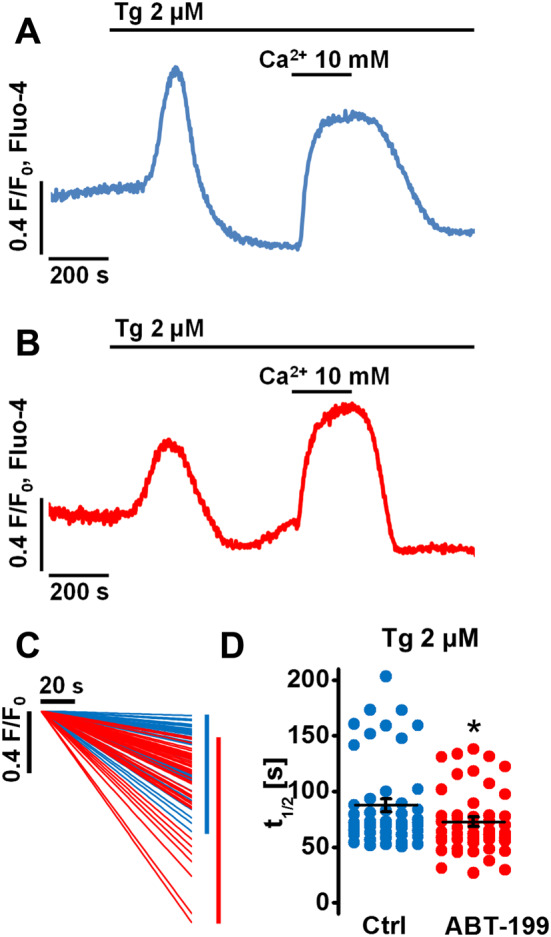
ABT‐199 affects cytosolic Ca^2+^ extrusion in PACs. *N*, number of independent repeats in the experiments; *n*, number of independent regions of interest in the experiment (see the text for details). (A) Sample [Ca^2+^]_i_ trace recorded in a PAC pre‐incubated for 2 h in the extracellular buffer (control). Application of Tg in the absence of external Ca^2+^ resulted in the depletion of the ER Ca^2+^ store. Subsequent exposure to an external solution containing 10 mM Ca^2+^ for a period of 200 s triggered store‐operated Ca^2+^ entry. Returning the PACs to a Ca^2+^‐free external solution again reduces elevated [Ca^2+^]_I_, which in the presence of Tg is due to Ca^2+^ extrusion across the plasma membrane. (B) Sample [Ca^2+^]_i_ trace recorded in a PAC pre‐incubated for 2 h with 1 μM ABT‐199. The same protocol was used as above in (A). (C) Linear fits calculated to the extrusion phases recorded in control (blue) and ABT‐199‐treated (red) cells (as in A and B). Scale bars: x axis, 20 s; and y axis, 0.4 F/F_0_ (Fluo‐4). (D) Dot chart shows the half‐times (t_1/2_) of cytosolic Ca^2+^ extrusion recorded for control (blue, *N* = 7, *n* = 48, 87.8 ± 5.7 s) and ABT‐199‐treated PACs (red, *N* = 7, *n* = 46, 72.7 ± 4.2 s) as shown in (A) and (B) respectively. Student's t‐test was applied for statistical analysis.

Since Bcl‐2 was previously found to regulate cytosolic Ca^2+^ extrusion in PACs by modulating plasma membrane Ca^2+^ ATPase (PMCA) activity (Ferdek *et al.,* 2012), we wanted to address whether pharmacological inhibition of Bcl‐2 by ABT‐199 could affect this process. In order to measure Ca^2+^ extrusion, a protocol similar to that described before (Ferdek *et al.,* 2012) was used. PACs pretreated for 2 h with 1 μM ABT‐199 or incubated for the same amount of time in extracellular buffer (control) were exposed to Tg, a potent SERCA inhibitor, in Ca^2+^‐free solution (Figure 6A, B). The application of Tg results in the emptying of the ER Ca^2+^ stores (manifested as the initial response in Figure 6A, B). Increasing extracellular Ca^2+^ concentration to 10 mM induced Ca^2+^ influx to the cytosol *via* store‐operated Ca^2+^ channels, which are opened upon ER Ca^2+^ depletion. Once a [Ca^2+^]_i_ plateau was established (the second response in Figure 6A, B), extracellular Ca^2+^ was removed again causing the [Ca^2+^]_i_ to decline towards the baseline levels. Since Ca^2+^ uptake into the ER is inhibited by Tg, the clearance of [Ca^2+^]_i,_ in Ca^2+^‐free solution reflects Ca^2+^ extrusion across the plasma membrane, mainly *via* PMCA. Despite a substantial variability in cytosolic Ca^2+^ extrusion rates in both control and ABT‐199‐treated cells, the apparent rate of extrusion was on average slightly faster in the presence of ABT‐199 (Figure 6A, B, sample traces). The distribution of the linear fits to the extrusion rates in ABT‐199‐treated cells was shifted towards those with faster declines (Figure 6C). This difference is well reflected by a lower t_1/2_ (72.7 ± 4.2 s) for ABT‐199‐treated PACs compared to control cells (87.7 ± 5.7 s), which translates into a shorter time required for the fluorescence of the Ca^2+^ indicator to decrease by half the difference between the [Ca^2+^]_i_ plateau and the baseline (Figure 6D). Although this effect is relatively minor and also much less prominent to that observed upon the complete loss of Bcl‐2 (Ferdek *et al.,* 2012), a potentiation of cytosolic Ca^2+^ extrusion may underlie the modest reduction of pathophysiological Ca^2+^ responses evoked by TLC‐S in the presence of ABT‐199 (Figure 4A).

## Discussion

It is very well established that the Bcl‐2 family proteins play a role in the regulation of intracellular Ca^2+^ homeostasis (Vervliet *et al.,* 2016). Although primarily associated with the mitochondrial membranes, these proteins also reside in other cell compartments such as the cytosol, the ER and the nuclear envelope (Krajewski *et al.,* 1993). At the ER (the main intracellular Ca^2+^ store), the anti‐apoptotic Bcl‐2‐family members regulate several proteins involved in Ca^2+^ signalling (Vervliet *et al.,* 2016), including the IP_3_R (Rong *et al.,* 2008, 2009; Ivanova *et al.,* 2014). The binding of Bcl‐2 and Bcl‐xL to the C‐terminal region of IP_3_R results in sensitization of the receptor and triggers pro‐survival Ca^2+^ oscillations (White *et al.,* 2005; Eckenrode *et al.,* 2010). Bcl‐2 also interacts with the central regulatory domain of IP_3_R *via* its BH4 domain thereby inhibiting excessive pro‐apoptotic Ca^2+^ release (Chen *et al.,* 2004; Rong *et al.,* 2009; Monaco *et al.,* 2012). Besides IP_3_Rs, the anti‐apoptotic Bcl‐2 proteins also regulate intracellular Ca^2+^ handling *via* RyRs (Vervliet *et al.,* 2014), SERCA (Kuo *et al.,* 1998) and Bax inhibitor‐1 (Xu *et al.,* 2008) at the ER, as well as PMCA (Ferdek *et al.,* 2012) and the mitochondrial voltage‐dependent anion channel (Arbel and Shoshan‐Barmatz, 2010). Therefore, agents that bind to and inhibit the Bcl‐2‐family members may affect some of the above interactions and indirectly modulate intracellular Ca^2+^ homeostasis.

Inhibition of the anti‐apoptotic Bcl‐2 members by the early generation BH3 mimetics, BH3I‐2′ and HA14‐1, was associated with global and sustained Ca^2+^ responses induced in normal mouse PACs and in the rat pancreatic cancer cell line AR42J (Gerasimenko *et al.,* 2010). These BH3 mimetic‐elicited responses were later shown to be dependent on Bax, but not Bak or Bcl‐2 (Ferdek *et al.,* 2017). Moreover, HA14‐1 was demonstrated to inhibit SERCA and deplete ER Ca^2+^ stores, causing the ER stress‐mediated cell death (Hermanson *et al.,* 2009; Akl *et al.,* 2013).

This study demonstrates that selective inhibition of Bcl‐2 by ABT‐199 neither triggers substantial intracellular Ca^2+^ release in PACs (Figure 1) nor affects Ca^2+^ responses elicited by endogenous agonists at physiologically relevant concentrations (Figures 2 and 3). Targeted inhibition of Bcl‐xL by A‐1155463 only had a minor effect on the intracellular Ca^2+^ homeostasis, occasionally inducing infrequent Ca^2+^ transients in PACs (Figure 1C). In contrast, inhibition of more than one Bcl‐2 family member *via* simultaneous application of ABT‐199 and A‐1155463 (Figure 1B) or by ABT‐737 (blocker of Bcl‐2, Bcl‐xL and Bcl‐w; Figure 1A) triggered sporadic intracellular Ca^2+^ rises in PACs or a prolonged Ca^2+^ plateau. This suggests that the toxic effects of BH3 mimetics on Ca^2+^ associated with the inhibition of a wide range of the anti‐apoptotic Bcl‐2 proteins could be avoided by selectively targeting only one family member, specifically Bcl‐2 itself (Figure 1E). Although it was previously shown that ABT‐199 does not alter intracellular Ca^2+^ signalling in cell lines, including Bcl‐2‐dependent DLBCL cancer cell models (Vervloessem *et al.,* 2017b), this study is one of the first few to extend that finding to non‐transformed primary cells. Given the toxic effects of early generation BH3 mimetics on PACs, it was particularly timely and relevant to scrutinize the impact of the newly approved drug ABT‐199 on physiological and pathophysiological signalling in the exocrine pancreatic system and thus to elucidate potential pancreatoxic effects (Figures 1–5).

ABT‐199 did not alter Ca^2+^ responses elicited by physiological or supramaximal concentrations of ACh (Figure 2). These responses are primarily initiated by IP_3_R‐mediated Ca^2+^ release from the ER and further amplified by RyRs (Wakui *et al.,* 1990; Cancela, 2001). Physiological CCK signalling, mediated by RyRs, was also essentially unaffected by ABT‐199 (Figure 3). A minor effect on CCK responses was only present at very high concentrations of the secretagogue. These results are in line with our previous work in cell models where we showed that binding to and regulation of IP_3_Rs and RyRs by Bcl‐2 is independent of its hydrophobic cleft (Vervliet *et al.,* 2015; Ivanova *et al.,* 2016), and thus, it is unlikely to be affected by BH3 mimetics that interact with this site.

Although inhibition of Bcl‐xL by A‐1155463 or pan‐inhibition of Bcl‐2/Bcl‐xL/Bcl‐w by ABT‐737 potentiated pathophysiological Ca^2+^ responses induced by TLC‐S or menadione, selective inhibition of Bcl‐2 by ABT‐199 did not (Figure 4). In fact, TLC‐S‐elicited Ca^2+^ responses were even reduced in the presence of ABT‐199 (Figure 4A). This might be due to a modest effect of ABT‐199 on cytosolic Ca^2+^ extrusion (Figure 6), which is almost exclusively dependent on PMCA activity in PACs (Petersen, 2003; Ferdek *et al.,* 2012). As shown previously, Bcl‐2 has been found to have an inhibitory effect on PMCA in PACs (Ferdek *et al.,* 2012). ABT‐199 appears to interfere with this function of Bcl‐2, thereby enhancing Ca^2+^ extrusion by alleviating Bcl‐2's inhibitory effect on PMCA.

The moderate suppression of pathological Ca^2+^ signals by ABT‐199 was reversed by A‐1155463, when PACs were pre‐incubated with both inhibitors before application of TLC‐S, indicating that the effects on Ca^2+^ associated with Bcl‐xL inhibition dominate over those triggered by inhibition of Bcl‐2 (Figure 4A). Since A‐1155463 interacts with the hydrophobic cleft of Bcl‐xL, this site appears to play a role in the regulation of intracellular Ca^2+^ signalling in response to pathophysiological stimulants. The mechanisms remain unclear, although it may relate to interference by these drugs of Bcl‐xL‐dependent regulation of Ca^2+^‐release systems like IP_3_Rs (Yang *et al.,* 2016).

Despite the aforementioned effects on pathophysiological Ca^2+^ responses induced by TLC‐S and menadione (Figure 4), neither of the inhibitors used in the study markedly affected cell death in PACs (Figure 5A,D). These results may suggest that the relatively small effects of ABT‐199, A‐115563 or ABT‐737 on Ca^2+^ homeostasis in PACs are insufficient to increase the sensitivity of normal cells to cell death inducers. This finding could be particularly relevant to the situation when a BH3 mimetic is used in combination with other chemotherapeutic agents. Such combined therapies are currently undergoing clinical trials (Ferdek and Jakubowska, 2017). It is important to note that with the conditions applied (1 μM ABT‐199 for 2 h) ABT‐199 was potent at inducing cell death in Bcl‐2‐dependent cancer cells, including the activated B‐cell like lymphoma cancer cell line Ri‐1, the germinal centre B‐cell‐like lymphoma cell line OCI‐LY‐1 and primary cells derived from CLL patients (Figure 5E,F).

In conclusion, we report that selective inhibition of Bcl‐2 by ABT‐199, apart from having a modest effect on cytosolic Ca^2+^ extrusion (Figure 6), does not either dramatically alter intracellular Ca^2+^ signals on its own (Figure 1) or in response to physiological (Figures 2 and 3) and pathophysiological stimuli in isolated PACs (Figure 4). As a consequence, ABT‐199 does not sensitize non‐transformed healthy PACs to cell death (Figure 5). Therefore, we conclude that since ABT‐199 does not substantially affect intracellular Ca^2+^ homeostasis or sensitize healthy PACs to inducers of cell death, it should be safe for the pancreas when used in therapy for leukaemia.

## Author contributions

G.B. conceived the study with further input of M.J., P.F. and T.V.; G.B., T.V. and P.F. coordinated the work. M.J., P.F., M.K., C.M. and T.V. performed the experiments. M.J. and P.F. performed the data analysis and made the figures. The manuscript was drafted by T.V., M.J., G.B. and P.F. All authors were involved in the interpretation of the data and critically revised and approved the manuscript for submission.

## Conflict of interest

The authors declare no conflicts of interest.

## Declaration of transparency and scientific rigour

This Declaration acknowledges that this paper adheres to the principles for transparent reporting and scientific rigour of preclinical research recommended by funding agencies, publishers and other organisations engaged with supporting research.
